# Supplementary material for: Reducing INDEL calling errors in whole genome and exome sequencing data
Source: Genome Med. 2014 Oct 28;6(10):89. doi: 10.1186/s13073-014-0089-z (PMC4240813; doi:10.1186/s13073-014-0089-z)
Supplement: Additional file 1: Figure S1. — Coverage distributions and fractions of the WGS-WES intersection regions. Figure S2. Coverage distributions and fractions of the WES-specific INDELs regions. Figure S3. Pair-wise base coverage relationship of INDEL called by both WGS and WES data. Figure S4. Characterization of the false discovery rate (FDR) based on validation data. Table S1. Mean depth coverage of WGS and WES data in different regions. Table S2. Mean coverage fractions of WGS and WES data in different regions. Table S3. Mean percentage and mean number of high-quality, moderate-quality, and low quality INDELs in each call set. Table S4. Mean percentages of high-quality INDELs partitioned by the following categories: homopolymer (A/C/G/T), other short tandem repeats (other STR), and non STR INDELs. Table S5. Mean fractions of low-quality INDELs partitioned by the following categories: homopolymer (A/C/G/T), other short tandem repeats (other STR), and non STR INDELs. Table S6. Number of INDELs in the WGS and WES data with multiple signatures partitioned by the following categories: homopolymer (A/C/G/T), other short tandem repeats (other STR), and non-STR INDELs. Table S7. Number of reads in the following four regions: Exonic targeted regions, WGS-WES intersection INDEL regions, WGS-specific INDEL regions, WES-specific INDEL regions. Table S8. Probabilities of seeing k or more INDELs in a given family assuming a binomial distribution. Table S9. Putative de novo exonic INDELs in these two families before and after applying filtering critiria. Note S1. Analysis of the effect of new filtering criteria on de novo INDEL calls. [file 13073_2014_89_MOESM1_ESM.docx]

Additional File 1

**Supplemental Figures**

**Supplemental Figure S1**

**
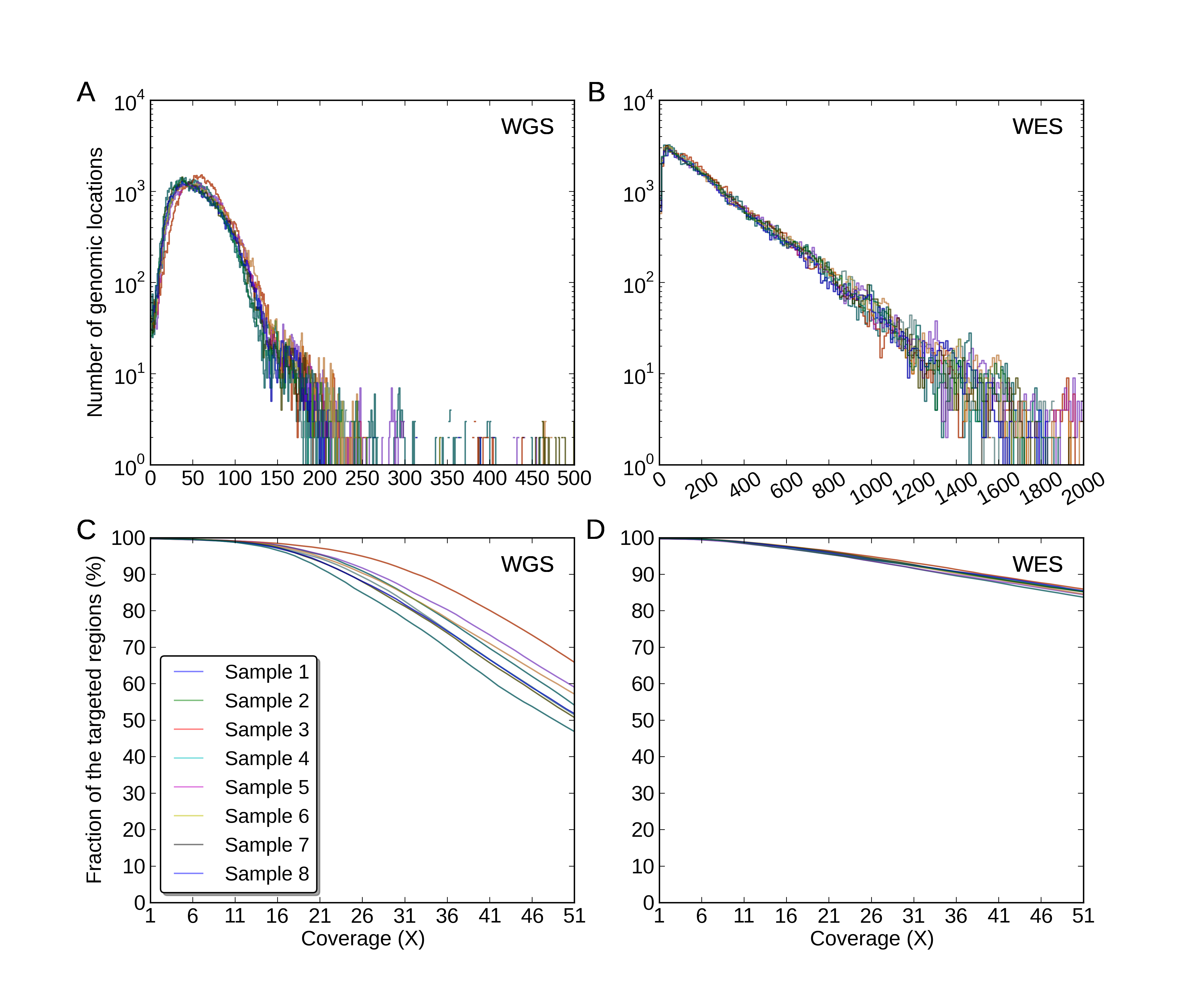
**

**Supplemental Figure S1.** **Coverage distributions and fractions of the WGS-WES intersection regions**. The coverage distributions of the WGS-WES intersection regions in (A) the WGS data, (B) the WES data. The Y-axis for (A) and (B) is of log10-scale. The coverage fractions of the WGS-WES intersection regions from 1X to 51X in (C) the WGS data, (D) the WES data.

**Supplemental Figure S2**

**
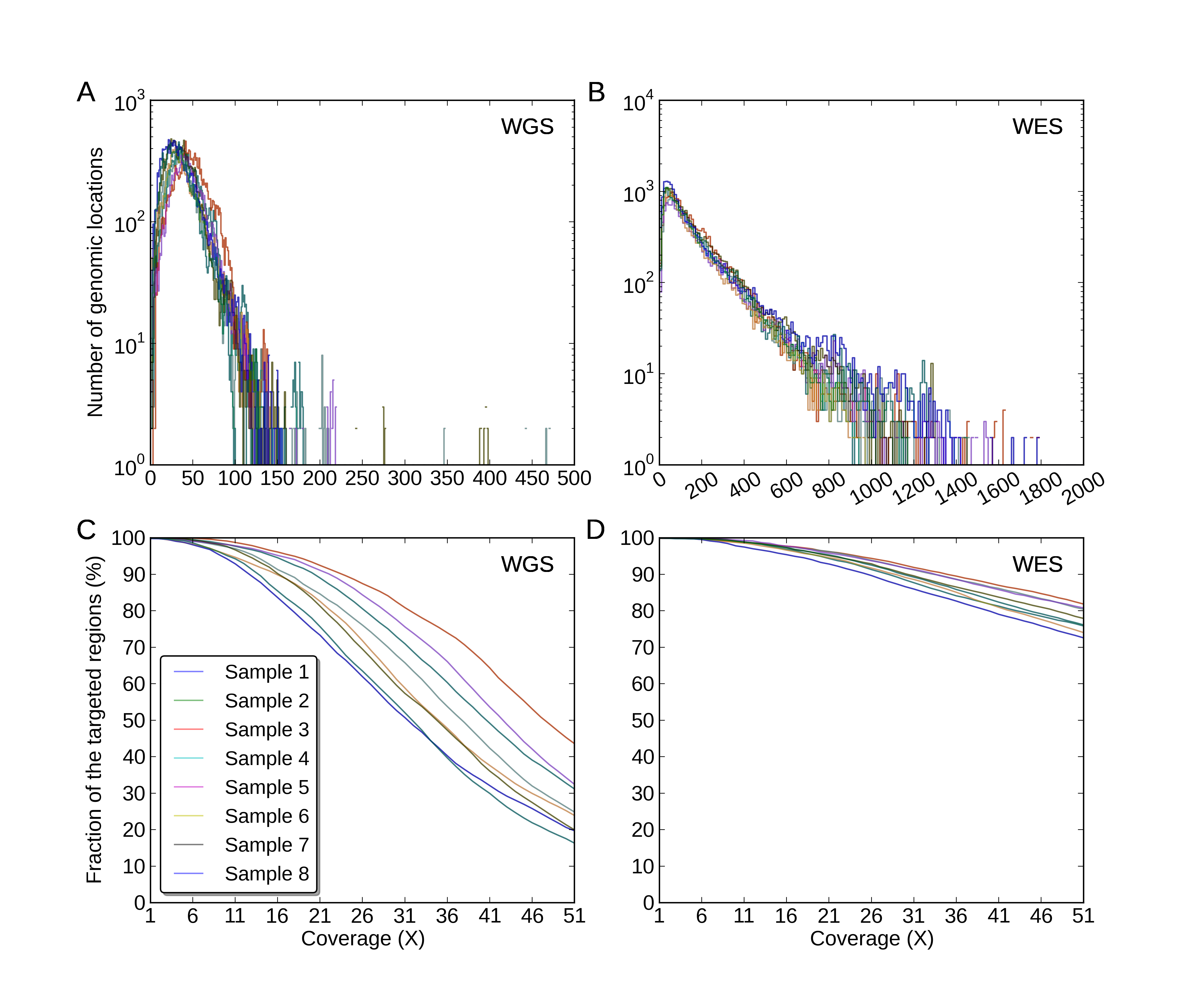
**

**Supplemental Figure S2.** **Coverage distributions and fractions of the WES-specific INDELs regions**. The coverage distributions of the WES-specific INDELs regions in (A) the WGS data, (B) the WES data. The Y-axis for (A) and (B) is of log10-scale. The coverage fractions of the WES-specific INDELs regions from 1X to 51X in (C) the WGS data, (D) the WES data.

**Supplemental Figure S3**

**
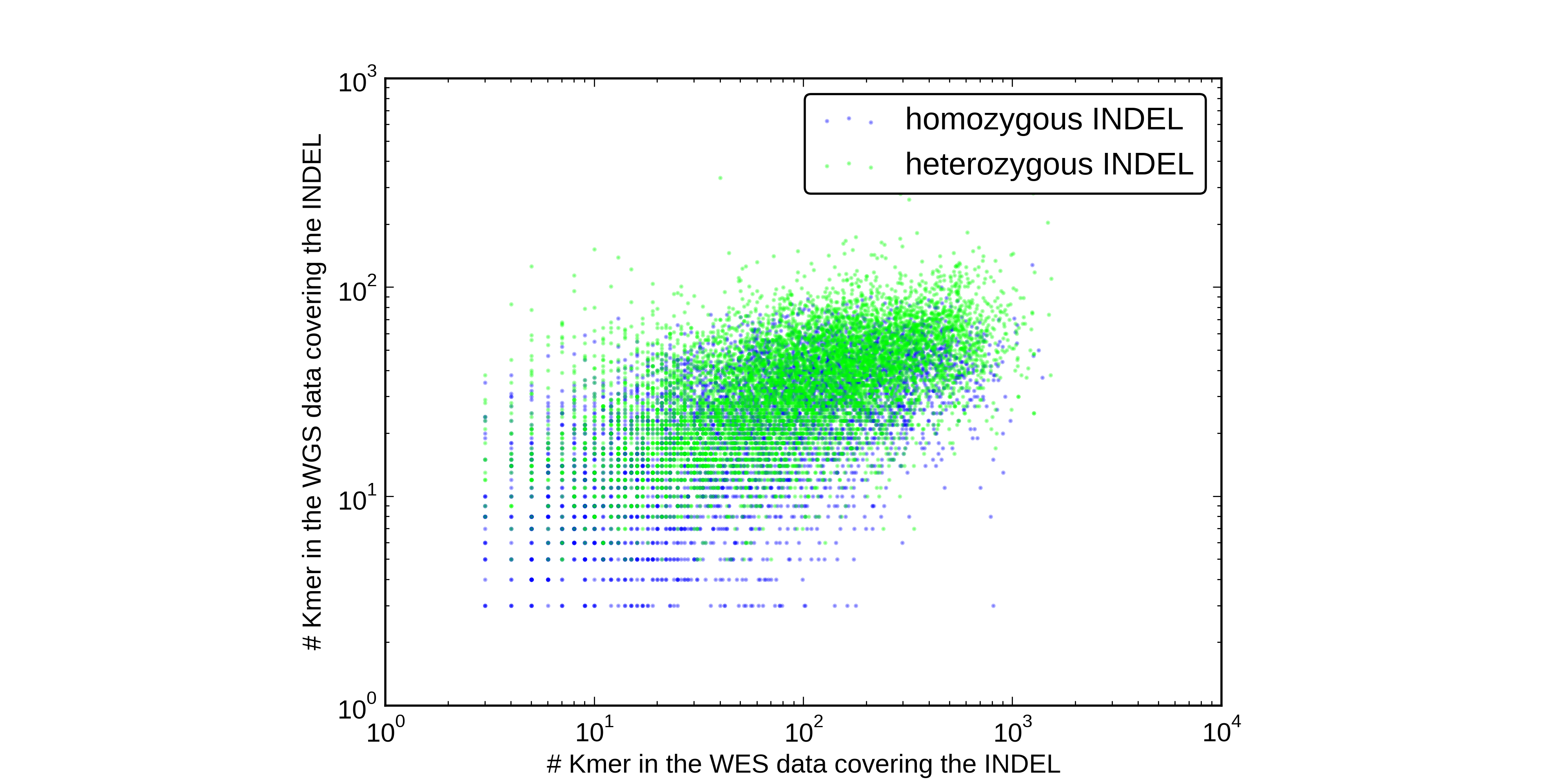
**

**Supplemental Figure S3.** **Pair-wise base coverage relationship of INDEL called by both WGS and WES data.** These INDELs were partitioned by zygosities: homozygous (blue) and heterozygous INDELs (green). The X-axis shows the number of k-mer covering an INDEL in the WES data, and the Y-axis shows the number for WGS data.

**Supplemental Figure S4**

**Supplemental Figure S4.** **Characterization of the false discovery rate (FDR) based on validation data.** INDELs were partitioned based on k-mer coverage of the alternative allele and the INDEL Chi-Square scores. The X-axis shows Chi-Square scores of INDELs less than a certain threshold, and the Y-axis represents the FDR.

**Supplemental Tables**

### Supplemental Table S1

**Supplemental Table S1.** **Mean** **depth** **coverage of WGS and WES data in different regions.** This table shows data corresponding to Figures 3 and 4, and Supplemenatal Figures S1 and S2. The standard deviation is shown in parentheses.

| Mean coverage | Exonic targeted regions | WGS-WES intersection INDEL regions | WGS-specific INDEL regions | WES-specific INDEL regions |
| --- | --- | --- | --- | --- |
| **WGS** | 71X (3.3X) | 58X (3.4X) | 61X (2.9X) | 41X (5.2X) |
| **WES** | 337X (18.2X) | 252X (7.0X) | 137X (12.1X) | 171X (10X) |

### Supplemental Table S2

**Supplemental Table S2.** **Mean** **coverage fractions of WGS and WES data in different regions.** This table shows data corresponding to Figures 3 and 4, and Supplemenatal Figures S1 and S2. The standard deviation is shown in parentheses.

|  | Coverage fraction | Exonic targeted regions | WGS-WES intersection INDEL regions | WGS-specific INDEL regions | WES-specific INDEL regions |
| --- | --- | --- | --- | --- | --- |
| WGS | \| 1X \| \| --- \| \| 20X \| \| 50X \| | \| 99.9% (0.1%) \| \| --- \| \| 98.2% (0.2%) \| \| 81.0% (3.1%) \| | \| 99.8%(0.04%) \| \| --- \| \| 96.0% (1.1%) \| \| 57.5% (6.0%) \| | \| 99.9%(0.03%) \| \| --- \| \| 93.9% (1.4%) \| \| 54.5% (0.4%) \| | \| 99.9%(0.06%) \| \| --- \| \| 86.9% (6.1%) \| \| 29.4% (9.4%) \| |
| WES | \| 1X \| \| --- \| \| 20X \| \| 50X \| | \| 83.9% (1.1%) \| \| --- \| \| 74.5% (0.1%) \| \| 72.0% (0.3%) \| | \| 99.8%(0.05%) \| \| --- \| \| 96.6% (0.3%) \| \| 85.7% (0.7%) \| | \| 55.8% (0.3%) \| \| --- \| \| 31.1% (2.1%) \| \| 25.2% (3.7%) \| | \| 99.9%(0.04%) \| \| --- \| \| 96.0% (1.0%) \| \| 78.7% (3.3%) \| |

### Supplemental Table S3

**Supplemental Table S3.** **Mean** **percentage and mean number of high-quality, moderate-quality, and low-quality INDELs in each call set.** This table shows data corresponding to Figure 5. The mean percentage and the mean number over eight samples are shown in the upper and the lower of a cell, respectively. The standard deviation is shown in parentheses.

|  | High quality | Moderate quality | Low quality |
| --- | --- | --- | --- |
| WGS-WES intersection INDELs | 89% (0.7%)  1454 (11.7) | 9% (0.5%)  148 (7.3) | 2% (0.5%)  31 (8.3) |
| WGS-specific INDELs | 78% (1.4%)  769 (13.9) | 15% (1.1%)  151 (10.7) | 7% (1.6%)  71 (15.8) |
| WES-specific INDELs | 22% (3.4%)  71 (11.2) | 37% (3.7%)  121 (11.9) | 41% (3.3%)  133 (10.9) |

### Supplemental Table S4

**Supplemental Table S4.** **Mean percentages of high-quality INDELs partitioned by the following categories: homopolymer (A/C/G/T), other short tandem repeats (other STR), and non-STR INDELs.** This table shows data corresponding to Figure 6. The standard deviation is shown in parentheses.

| Regions | WGS-WES intersection INDELs | WGS-specific INDELs | WES-specific INDELs |
| --- | --- | --- | --- |
| Poly-A | 11.2% (0.8%) | 13.6% (0.6%) | 24% (3.0%) |
| Poly-C | 0.09% (0.06%) | 0.3% (0.1%) | 1.4% (1.0%) |
| Poly-G | 0.3% (0.09%) | 0.5% (0.1%) | 0.6% (0.8%) |
| Poly-T | 9.0% (0.6%) | 7.9% (0.7%) | 30% (3.5%) |
| Other STR | 9.6% (0.5%) | 11.1% (0.9%) | 12.5% (3.1%) |
| Non-STR | 70% (1.2%) | 67% (1.1%) | 31.9% (6.1%) |

### Supplemental Table S5

**Supplemental Table S5.** **Mean fractions of low-quality INDELs partitioned by the following categories: homopolymer (A/C/G/T), other short tandem repeats (other STR), and non-STR INDELs.** This table shows data corresponding to Figure 6. The standard deviation is shown in parentheses.

| Regions | WGS-WES intersection INDELs | WGS-specific INDELs | WES-specific INDELs |
| --- | --- | --- | --- |
| Poly-A | 19.6% (12.5%) | 26.1% (7.0%) | 41.5% (3.2%) |
| Poly-C | 0.6% (1.6%) | 0% (0%) | 0.3% (0.3%) |
| Poly-G | 0% | 0.4% (0.09%) | 0.3% (0.3%) |
| Poly-T | 24.6% (11.2%) | 19.0% (5.6%) | 41.1% (3.5%) |
| Other STR | 21.0% (7.3%) | 11.9% (3.8%) | 6.2% (1.2%) |
| Non-STR | 34.1% (14.1%) | 42.6% (9.4%) | 10.7% (2.5%) |

**Supplemental Table S6**

**Supplemental Table S6.** **Number of INDELs in the WGS and WES data with multiple signatures partitioned by the following categories: homopolymer (A/C/G/T), other short tandem repeats (other STR), and non STR INDELs.** This table shows data corresponding to Figure 7. The standard deviation is shown in parentheses.

| Regions | WGS | WES |
| --- | --- | --- |
| Poly-A | 25 (5.7) | 35 (6.0) |
| Poly-C | 0.3 (0.4) | 1 (0.3) |
| Poly-G | 0.6 (0.5) | 0.8 (0.7) |
| Poly-T | 16 (4.0) | 36 (5.3) |
| Other STR | 25 (4.8) | 24 (3.4) |
| Non-STR | 9 (2.6) | 6 (1.4) |

### Supplemental Table S7

**Supplemental Table S7.** **Number of reads in the following four regions: Exonic targeted regions, WGS-WES intersection INDEL regions, WGS-specific INDEL regions, WES-specific INDEL regions.**

| Number of reads | Exonic targeted regions | WGS-WES intersection INDEL regions | WGS-specific INDEL regions | WES-specific INDEL regions |
| --- | --- | --- | --- | --- |
| WGS | 241,984 | 49,008 | 26,417 | 1,775 |
| WES | 815,945 | 205,698 | 44,346 | 11,251 |

**Supplemental Table S8**

**Supplemental Table S8.** **Probabilities of seeing k or more INDELs in a given family assuming a binomial distribution.** Here we assumed a binomial distribution of the *de novo* exonic INDELs in the 343 SSC families.

| Number of INDELs | = 0 | ≥ 1 | ≥ 2 |
| --- | --- | --- | --- |
| Probability | 0.78 | 0.22 | 0.03 |
| Number of INDELs | ≥ 3 | ≥ 4 | ≥ 5 |
| Probability | 0.0020 | 0.0001 | 0.000005 |

### Supplemental Table S9

**Supplemental Table S9.** **Putative *de novo* exonic INDELs in these two families before and after applying filtering criteria.** The number of INDELs within regions of homopolymer A (poly-A), homopolymer T (poly-T), and microsatellites (ms) are shown in parentheses.

| Putative *de novo* | WGS  (poly-A, poly-T, ms) | WES  (poly-A, poly-T, ms) | WGS  (After filtering) | WES  (After filtering) |
| --- | --- | --- | --- | --- |
| Family 1 | 45 (27, 14, 4) | 5 (3, 1, 1) | 0 | 0 |
| Family 2 | 49 (24, 22, 3) | 17 (8, 5, 4) | 0 | 0 |

**Supplemental Note 1**

**Analysis of the effect of new filtering criteria on *de novo* INDEL calls**

The two families in this study were previously reported in a population-scale autism study, with Sanger validation of *de novo* calls [[1](#_ENREF_1)]. We used the *de novo* mode of Scalpel to identify *de novo* INDELs in these two families again, resulting in one *de novo* call set for WGS data and another *de novo* call set for WES data per family. We partitioned each call set by regions and filtered out the low quality INDELs. Iossifov *et al.* reported a total of N = 85 *de novo* exonic INDELs in 343 families, that is, there was 0.1 *de novo* exonic INDEL per child [1]. If we assume a binomial distribution of the *de novo* exonic INDELs with an equal chance (*P* = 1/343), the probability of seeing at least X *de novo* exonic INDELs in a given family in this study can be computed as below:

$$P\left( X\geq k INDELs \right)= 1-\sum_{0}^{k} P\left( X=k-1 \right)=1-\sum_{0}^{k-1} \binom{N}{X}p^{X}q^{N-X}$$

where P( X ≥ k ) is the probability of a given family having k or more *de novo* INDELs; N is the total number of exonic *de novo* INDELs reported, that is, N = 85; p is the probability of a hit on a given trial, that is, p = 1/343; q = 1-p.

**Applications of using filtering criteria to reduce false positive *de novo* INDELs**

Supplemental Table S8 shows the probabilities of seeing more than K INDELs from one of the 343 families reported in Iossifov *et al.* [[1](#_ENREF_1)]. Scalpel has a *de novo* analysis mode; it could re-assemble each region associated with the candidate INDELs across the family members using a more sensitive parameter setting. This setting was indeed more sensitive for detecting *de novo* INDELs than single-sample calling. Due to this, we used the following more rigorous filtering criteria than the above assessment to exclude any spurious false-positive *de novo* INDELs: coverage of the alternative allele >10 and Chi-Square score <4. Supplemental Table S9 showed the number of putative *de novo* INDELs in two families before and after applying this filtering criteria. All of the spurious *de novo* varaints in the two families were successfully excluded, which was consistent with the validated results in the variant database reported by Iossifov *et al.* [[1](#_ENREF_1)]. We noticed that, in both families, the majority of these false positive *de novo* INDELs were poly-A/T relevant (91% for WGS, 78% for WES), which was consistent with the above assessment. This suggested that if we used very sensitive callers, we should control for poly-A/T false positive *de novo* INDELs by applying a more rigorous filtering criteria, especially in population-scale sequencing projects, where there is substantial expense with experimental validation.

**References**

1. Iossifov I, Ronemus M, Levy D, Wang Z, Hakker I, Rosenbaum J, Yamrom B, Lee YH, Narzisi G, Leotta A, Kendall J, Grabowska E, Ma B, Marks S, Rodgers L, Stepansky A, Troge J, Andrews P, Beritsky M, Pradhan K, Ghiban E, Kramer M, Parla J, Demeter R, Fulton LL, Fulton RS, Magrini VJ, Ye K, Darnell JC, Darnell RB, *et al*: **De novo gene disruptions in children on the autistic spectrum.** *Neuron* 2012, **74:**285–299.
